# Physicochemical and Mechanical Properties of Non-Isocyanate Polyhydroxyurethane (NIPHU) Films with Silver Nanoparticles (AgNPs)

**DOI:** 10.3390/ma19214698

**Published:** 2026-11-04

**Authors:** Emmanuel Cano Depablos, Ana Velez-Pardo, Luis Eduardo Díaz, Nestor Sanchez, Manuel F. Valero

**Affiliations:** 1 Master’s Program in Process Design and Management, Faculty of Engineering, Universidad de La Sabana, Chia 140013, Colombia; emmanuelcanpa@unisabana.edu.co (E.C.D.); anavepar@unisabana.edu.co (A.V.-P.); 2 Energy, Materials and Environment Research Group (GEMA), Faculty of Engineering, Universidad de La Sabana, Chia 140013, Colombia; nestor.sanchez1@unisabana.edu.co; 3 InnovaCiencia CB Research Group, Faculty of Sciences, Universidad Colegio Mayor de Cundinamarca, Bogota 110311, Colombia; leduardodiaz@universidadmayor.edu.co

**Keywords:** non-isocyanate polyhydroxyurethane, bio-based polymers, soybean oil, silver nanoparticles, mechanical properties

## Abstract

**Highlights:**

**What are the main findings?**

- Functionalization routes influenced silver incorporation and NIPHU film behavior.
- Ag-containing functionalization was associated with increased wettability and mechanical resistance.
- ST-4-30 exhibited the highest total silver incorporation among the evaluated formulations.
- Functionalization routes produced distinct surface heterogeneity patterns.

**What are the implications of the main findings?**

- Biological and chemical synthesis routes produce distinct silver incorporation profiles.
- Plant extracts are feasible reducing systems for AgNP functionalization.
- *Acca sellowiana* peel can be used as a reducing agent for AgNP synthesis.

**Abstract:**

Bio-based non-isocyanate polyhydroxyurethanes (NIPHUs) provide a versatile platform for functional polymeric materials while reducing dependence on conventional isocyanate-based polyurethane synthesis. In this work, soybean oil-derived NIPHU films were functionalized with silver nanoparticles (AgNPs) synthesized by biological routes using *Solanum tuberosum* and *Acca sellowiana* peel extracts, and by chemical reduction with sodium borohydride (NaBH_4_). This study was aimed at determining how formulation conditions affected AgNP formation, silver incorporation and the resulting physicochemical and mechanical properties. Plant extracts were characterized by phenolic compounds, reducing sugars and antioxidant activity, while AgNP formation was assessed by UV-vis spectroscopy, X-ray diffraction and ATR-FTIR. Functionalized films were characterized by wettability, mechanical performance, thermal stability, fluid absorption, total silver content and surface morphology. All routes enabled the formation of metallic AgNPs, while the films exhibited differences in silver incorporation and mechanical performance. The functionalized films generally exhibited increased wettability and mechanical resistance compared with the unfunctionalized NIPHU, whereas phosphate-buffered saline (PBS) uptake did not consistently increase and thermal stability of the NIPHU matrix was maintained. ST-4-30 showed the most balanced mechanical and physicochemical response. Overall, the AgNP synthesis route emerged as an effective formulation variable for tuning the properties of soybean oil-derived NIPHU films.

## 1. Introduction

In materials design, polymers constitute a fundamental platform because of their chemical composition, molecular architecture, and intermolecular interactions, which can be modified to control their macroscopic properties [1,2]. Among polymers, polyurethanes (PUs) stand out due to the versatility of their segmented structure, in which the nature and proportion of hard and soft segments, with hydrogen bonding interactions associated with urethane groups, enable the modulation of mechanical properties, thermal behavior, molecular mobility, and surface characteristics [3,4]. This adaptability has attracted considerable interest in biomedical applications because it enables their flexibility, mechanical resistance, biocompatibility, and structural versatility [5] by enabling the preparation of materials ranging from rigid networks to deformable films and elastomers through changes in polyol and isocyanate chemistry, chain extenders, monomer functionality, and modifying agents [3,6,7].

Structural modification of PUs has been developed by changing their molecular constituents and by incorporating secondary phases or modifying agents capable of establishing new interactions with the polymer matrix. Among the modifiers investigated are graphene and graphene oxide [8,9,10], ceramic and oxide nanoparticles such as TiO_2_ [11], nanolignin [12], and metallic nanoparticles such as silver nanoparticles [5]. These modifiers have been incorporated either as dispersed fillers, hybrid phases, or surface-associated components. Nevertheless, conventional PU synthesis relies on isocyanates, compounds associated with toxicity concerns and dependence on petrochemical resources [7,13,14], which has driven the development of alternative routes. Among these alternatives, non-isocyanate polyurethanes (NIPUs), particularly polyhydroxyurethanes (PHUs) obtained by cyclic carbonate aminolysis, have emerged as a promising alternative, enabling urethane linkage formation without isocyanates while introducing hydroxyl groups into the polymer backbone, broadening their potential applications [7].

Research on NIPUs has increasingly focused on vegetable oil-derived systems, which can produce networks with markedly different mechanical and thermal responses. Catalá et al. showed that crosslinking density can be used to tailor the behavior of bio-based NIPUs [15]; Centeno-Pedrazo et al. used carbonated soybean oil to obtain NIPU systems suitable for coating with performance comparable to conventional formulations [16]; and Patel et al. showed that diamine chain length and curing time modify the mechanical and thermal properties of soybean oil-derived NIPU films [17]. Moreover, Raftopoulos et al. further demonstrated that precursor structure and hydrogen bonding interactions affect segmental mobility [18], while other vegetable oil-based systems have confirmed that network chemistry enables the preparation of NIPUs with tunable mechanical and thermal properties [19,20,21]. Overall, these investigations indicate that material performance arises from combined effects of composition, process conditions and precursor characteristics.

Nanoparticle incorporation has been explored for modifying internal interactions within a polymer matrix and its macroscopic behavior. AgNPs are of particular interest because the characteristics of nanomaterials depend on the conditions for silver reduction, nucleation, growth, and stabilization [22]. These nanoparticles can be synthesized through chemical [5,23], physical [24,25] or biological routes [26,27,28,29]; however, the synthesis pathway strongly influences efficiency, toxicity, and environmental impact [22,30]. Biological synthesis using plant-derived extracts has gained relevance as an alternative to conventional reducing agents such as sodium borohydride, used because of its ability to reduce silver ions [31]. Nanoparticle incorporation has been evaluated in PU matrices [11]; however, its incorporation in soybean oil-derived NIPHU films remains unexplored, particularly regarding how the synthesis route influences physicochemical and mechanical properties.

Different plant matrices have been investigated as reducing and stabilizing agents for the biological synthesis of AgNPs. Accordingly, matrices such as *Moringa oleifera* leaves [32], *Arctium lappa* leaves and flowers [33], *Solanum tuberosum* peels [34], *Coriandrum sativum* stems [28], purple potato (*Solanum tuberosum* INIA 328—Kulli papa) anthocyanin extracts [35], *Zea mays* silk [36], and *Musa paradisiaca* peels [37] have been used for nanoparticle synthesis. These studies show differences in phytochemical composition that can modify nanoparticle nucleation and stabilization, affecting particle size, crystallinity, and spectroscopic characteristics [28,32,33,35]. For example, *M. oleifera* leaf extract produced predominantly spherical AgNPs with an average diameter of 10.0 ± 6.0 nm approximately [32], whereas *S. tuberosum* peel extract yielded stable AgNPs with sizes ranging from 3.91 nm to 27.07 nm [34]; purple potato extract has also been shown to act simultaneously as a reducing and a stabilizing system, producing AgNPs with 21.6 nm of diameter and a face-centered cubic crystalline structure [35], while *M. paradisiaca* peel-derived diols have been associated with silver reduction and the formation of face-centered cubic nanoparticles [37]. In this context, *Acca sellowiana* peel is particularly interesting because of its rich source of phenolic compounds and substantial antioxidant capacity [38,39]. Nevertheless, its use as a reducing system for AgNP synthesis remains underexplored, while the valorization of its peel could provide an additional strategy for the use of fruit processing biomass.

Despite advances in both bio-based NIPUs, particularly non-isocyanate polyhydroxyurethanes (NIPHUs) and silver-containing PU nanocomposites [15,17], information relating the AgNP synthesis route to the resulting properties of a soybean oil-derived NIPHU matrix remains insufficiently explored. Previous studies have demonstrated that the polymer structure, diamine selection, crosslinking density, and process conditions can influence the mechanical, thermal and fluid interaction properties [15,17,18]. In parallel, studies in AgNP synthesis have shown that the reducing agent and synthesis conditions may influence nanoparticle formation and final physicochemical characteristics [22,34]. Once incorporated into PU matrices, AgNPs may also modify mechanical, thermal and surface properties through nanoparticle–polymer interactions [40]. Nevertheless, studies integrating these aspects by comparing AgNP synthesis routes within a bio-based NIPHU matrix remain scarce.

The principal contribution of the present study is the incorporation of AgNPs obtained through different reduction systems into a single soybean oil-derived NIPHU matrix, allowing route-dependent differences in the resulting functionalized films to be assessed while maintaining the polymeric chemistry. In addition, two plant extracts based on *Solanum tuberosum* and *Acca sellowiana* peels were compared with conventional NaBH_4_ reduction, introducing an agro-industrial residue valorization approach and extending the investigation of *A. sellowiana* as an underexplored reducing system. Therefore, this study aimed to develop and characterize soybean oil-derived non-isocyanate polyhydroxyurethane films with AgNPs obtained through biological routes using *Solanum tuberosum* and *Acca sellowiana* extracts and through chemical reduction using NaBH_4_, evaluating the resulting structural, mechanical, thermal, and fluid interaction properties.

## 2. Materials and Methods

### 2.1. Synthesis of Non-Isocyanate Polyhydroxyurethanes (NIPHUs)

Carbonated soybean oil (CSBO) was synthesized using a methodology adapted from a previous report [41]. Epoxidized soybean oil (ESBO) and tetrabutylammonium bromide (TBABr) as catalyst (5 mol% relative to epoxide content) were introduced into a BR-HS high-pressure reactor (Berghof Products, Eningen unter Achalm, Germany), heated to 105 °C, and CO_2_ was supplied at 6.0 bar for 24 h under stirring at 850 rpm, then cooled to 25 °C. NIPHUs were prepared following the procedure described in [41]. CSBO was heated to 60 °C and reacted with 1,4-butanediamine (BDA) and 1,3-cyclohexanebis(methylamine) (CHM) at a 50:50 molar ratio, using a cyclic carbonate-to-amine ratio of 1:2. After 5 min, the mixture was poured into molds, cured at 90 °C for 12 h, and immersed in distilled water for 48 h.

### 2.2. Production of Plant Extracts from Agro-Industrial Residues

Extracts from *Acca sellowiana* (AS) and *Solanum tuberosum* (ST) peels were obtained following an adapted methodology from [34]. Peels were disinfected with NaOCl (0.01% *w*/*v*) for 2 min, rinsed, and dried at 40 °C for 48 h before grinding into powder. Extraction used a 3:30 g solid-to-liquid ratio in distilled water at 80 °C (ST) and 40 °C (AS) for 40 min. Suspensions were filtered through sterile gauze and a 0.45 μm membrane, then lyophilized using a FreeZone™ stoppering tray freeze dryer model (Labconco Corporation, Kansas City, MO, USA) for 48 h and stored at 4 °C.

### 2.3. Synthesis of Silver Nanoparticles (AgNPs) in Suspension

AgNP synthesis was carried out in an aqueous suspension using polyvinylpyrrolidone (PVP, 3% *w*/*v*, 360 mg) as the stabilizing agent [42]. AgNO_3_ was added at concentrations listed in Table 1 and stirred at 500 rpm for 10 min. The reducing agent was added dropwise at a 5:1 AgNO_3_-to-reducing-agent volume ratio at 600 rpm. Plant extract-mediated syntheses were adjusted to pH 9.0 with 0.1 M NaOH and reacted for 2 h; NaBH_4_-mediated synthesis was conducted in an ice bath (2–10 °C) for 30 min.

### 2.4. Preparation of Functionalized Films (NIPHU-AgNPs)

**Figure 1:**
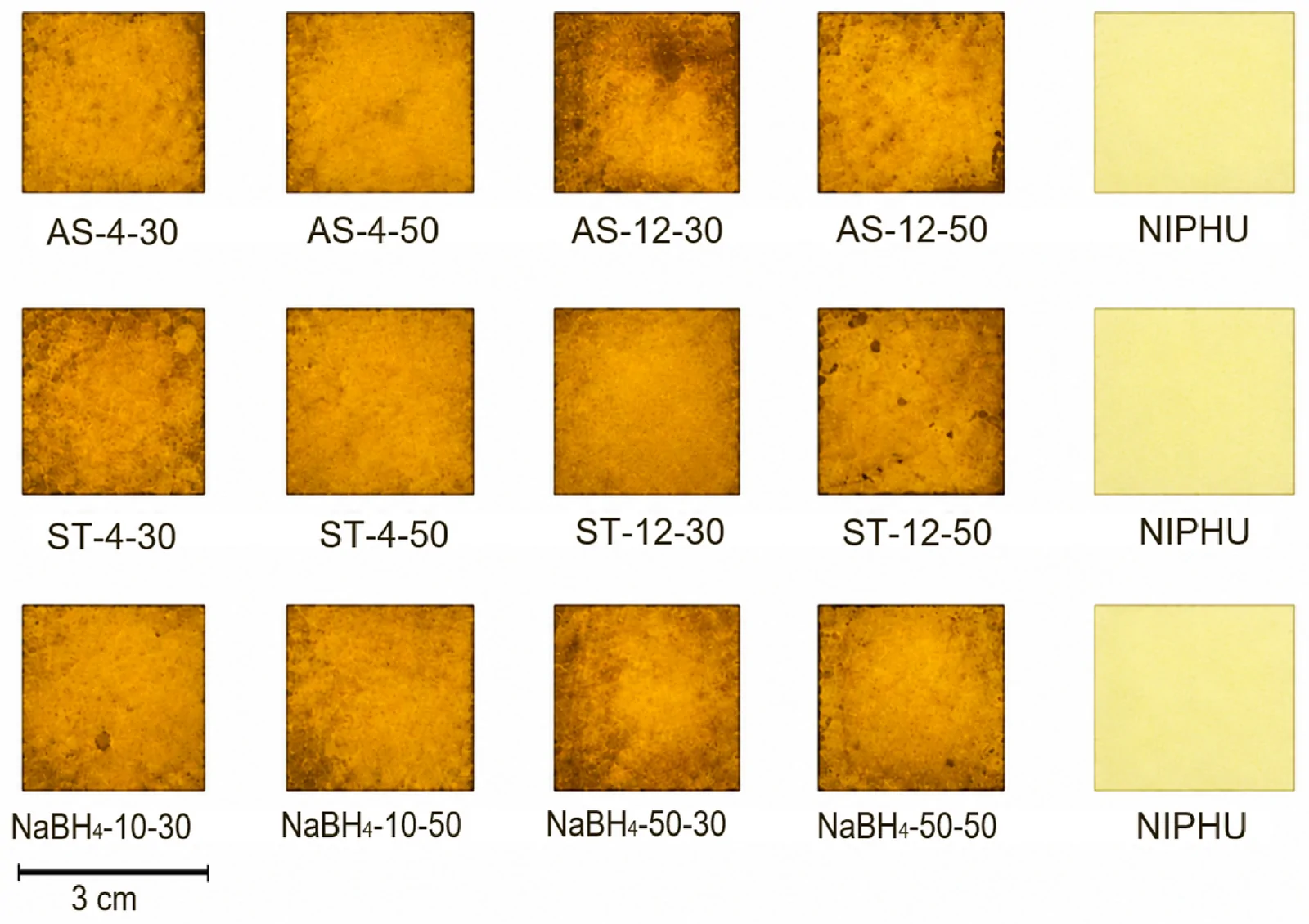
Visual appearance of NIPHU films synthesized with silver nanoparticles using *Acca sellowiana* (AS), *Solanum tuberosum* (ST), and NaBH_4_ as reducing systems. Films are labeled according to the treatment conditions. NIPHU corresponds to the film without functionalization.

Film functionalization followed Ashraf et al. [43]. Square films (6 cm × 6 cm) were immersed in a 20 mL AgNO_3_ solution for 4 h, then the reducing agent was added dropwise (5:1 AgNO_3_-to-reducing agent volume ratio). Reducing agents and AgNO_3_ concentrations evaluated are summarized in Table 1. Plant extract treatments were adjusted to pH 9.0 with 0.1 M NaOH for 2 h; NaBH_4_ treatments were conducted in an ice bath (2–10 °C) for 30 min. Films were washed with distilled water and dried at 60 °C for 1 h. The visual appearance of the resulting NIPHU-AgNP films obtained is shown in Figure 1.

### 2.5. Characterization

Lyophilized extracts were characterized for total phenolic content, reducing sugars, and antioxidant activity using calibration curves with gallic acid, glucose, and trolox as standards, measuring absorbance at 750 nm and 515 nm, with an iMark™ microplate reader (Bio-Rad Laboratories, Inc., Hercules, CA, USA). Structural characteristics of ESBO, CSBO, NIPHUs, extracts, and AgNPs were assessed by ATR-FTIR (Cary 630 FTIR, Agilent Technologies Inc., Santa Clara, CA, USA) over the range of 400–4000 cm^−1^. Molecular structures of products of carbonation reactions were analyzed via nuclear magnetic resonance (NMR) to obtain ^1^H NMR and ^13^C NMR spectra using a Bruker Avance Neo 400 MHz spectrometer (Bruker BioSpin Corporation, Billerica, MA, USA) and deuterated chloroform (CDCl_3_) as the solvent. AgNPs were further characterized by UV-Vis spectroscopy (Helios Omega UV-Vis spectrophotometer, Thermo Fisher Scientific Inc., Waltham, MA, USA) over a wavelength range of 350–750 nm) and X-ray diffraction (XRD) (Aeris X-ray diffractometer, Malvern Panalytical, Almelo, The Netherlands).

### 2.6. Water Contact Angle

The hydrophilic character of NIPHU–AgNP films was assessed by the sessile drop method using an OCA20 instrument (Data Physics, Filderstadt, Germany) with deionized water at 25 °C [41].

### 2.7. Tensile Mechanical Testing

Maximum stress, elongation at break, and secant modulus were determined using a DMA 850 instrument (TA Instruments, New Castle, DE, USA) following an adaptation of ASTM D638-10 [44], with a 0.5 N load cell at 25 mm min^−1^ [41]. Three specimens (20 mm × 5 mm) were evaluated per treatment. The thickness of each specimen was measured prior to tensile testing and used for the calculation of the cross-sectional area.

### 2.8. Thermogravimetric Analysis and Differential Scanning Calorimetry (DSC)

Thermal stability was assessed by TGA/DTG from 25 to 600 °C under nitrogen at 25 °C min^−1^ [41]. Disc-shaped samples (6 mm diameter) were evaluated. DSC measurements were performed using a DSC 3+ analyzer (Mettler Toledo, Schwerzenbach, Switzerland) over a temperature range from −70 to 150 °C under a nitrogen atmosphere (20 mL min^−1^), at a heating rate of 20 °C min^−1^, using disc-shaped samples (6 mm diameter). The glass transition temperature (T_g_) was estimated from the inflection point of the DSC heat flow curve within the selected glass transition region. For each experimental point, the heat flow derivative was calculated using a centered finite difference approximation, according to Equation (1).

(1) $$\frac{\mathrm{dHF}}{\mathrm{dT}}\approx \left({\mathrm{HF}}_{i+1}-{\mathrm{HF}}_{i-1}\right)/\left(T_{i+1}-T_{i-1}\right)$$

The T_g_ value was assigned to the temperature corresponding to the maximum absolute derivative according to Equation (2).

T_g_ = T[max(|dHF/dT|)] (2)

### 2.9. PBS 1X and Water Absorption

PBS and water absorption (%) were evaluated using disc-shaped samples (6 mm diameter) incubated at 37 °C, following an adaptation of ISO 62:2008 [45] in phosphate-buffered saline (PBS 1X: 137 mM NaCl, 10 mM phosphate, 2.7 mM KCl) and distilled water at a 1 cm^2^:10 mL surface area-to-volume ratio. Measurements were taken at 0, 4, 17, 42, and 114 h.

### 2.10. Total Silver Content

The total silver content in NIPHU–AgNP films was quantified by inductively coupled plasma optical emission spectrometry (ICP-OES) using an iCAP 6300 Duo instrument (Thermo Scientific, Cambridge, UK). Samples of known mass were digested with concentrated HNO_3_ until a homogeneous solution was obtained.

### 2.11. Scanning Electron Microscopy (SEM)

Surface morphology was evaluated by scanning electron microscopy (SEM) using a Quanta 200 microscope (FEI Company, Hillsboro, OR, USA). Images were acquired at magnifications of 100× and 2000× using an accelerating voltage (HV) of 20 kV and a backscattered electron (BSE) detector. Prior to analysis, the samples were sputter-coated using a Q150R ES sputter coater/carbon evaporator (Quorum Technologies Ltd., Laughton, East Sussex, UK) with Au/Pd (60/40) coating at a sputtering current of 20 mA for 60 s. The images were used to assess the surface characteristics, morphology, and distribution of surface features present in the NIPHU films analyzed.

### 2.12. Statistical Analysis

Data are reported as mean ± standard deviation (SD). Differences among treatments were evaluated by one-way ANOVA followed by Tukey’s test (*p* < 0.05); data were transformed when required to satisfy model assumptions. ICP-OES data were analyzed by factorial permutation ANOVA using the Freedman–Lane procedure, followed by a studentized permutation max-T post hoc test with FWER control.

### 2.13. Use of Artificial Intelligence-Assisted Tools

For the preparation of this work, the authors used OpenAI ChatGPT (GPT-6; OpenAI, San Francisco, CA, USA) as an assistive tool for language refinement, improvement of clarity and organization. It was also used as support in the development and debugging of scripts for statistical analysis and figure formatting. All AI-assisted text, code and outputs were verified and edited by the authors as needed. The authors take full responsibility for the accuracy, interpretation and final content of the publication.

## 3. Results and Discussion

### 3.1. Characterization of Plant Extracts

The characterization results of the lyophilized ST and AS extracts are presented in Table 2. AS showed significantly higher total phenolic content, reducing sugars, and antioxidant activity compared with ST. The presence of these compounds in both extracts suggests their involvement in Ag^+^ reduction and AgNP stabilization during biological synthesis, as they can act simultaneously as reducing and capping agents [34]. Additionally, the use of agro-industrial residues provides an alternative route for biomass valorization while avoiding the direct use of whole plant material.

### 3.2. Attenuated Total Reflectance Fourier Transform Infrared Spectroscopy (ATR-FTIR)

**Figure 2:**
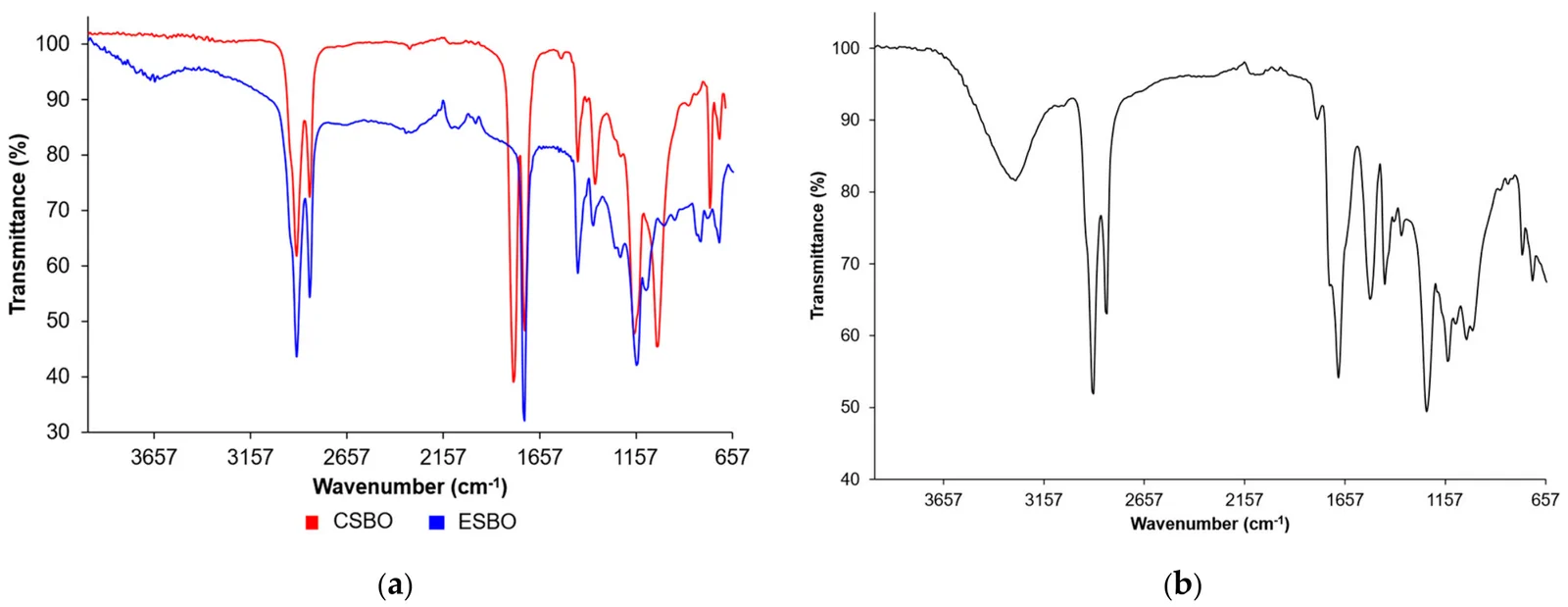
(**a**) ATR-FTIR spectra for ESBO (blue) and CSBO (red); (**b**) ATR-FTIR spectra for NIPHU films.

ATR-FTIR spectra of ESBO, CSBO, and NIPHUs are shown in Figure 2. Conversion of epoxy groups into cyclic carbonates was confirmed by the disappearance of the epoxy band at 834 cm^−1^ and the appearance of signals at 1046, 1200, and 1801 cm^−1^ (Figure 2a). NIPHU formation was evidenced by the disappearance of the 1801 cm^−1^ band and the emergence of peaks at 1689, 1536, and 3309 cm^−1^, corresponding to urethane carbonyl stretching, C–N stretching/N–H bending, and N–H/OH stretching vibrations, respectively (Figure 2b), confirming successful polymer synthesis [41].

### 3.3. Nuclear Magnetic Resonance (NMR)

**Figure 3:**
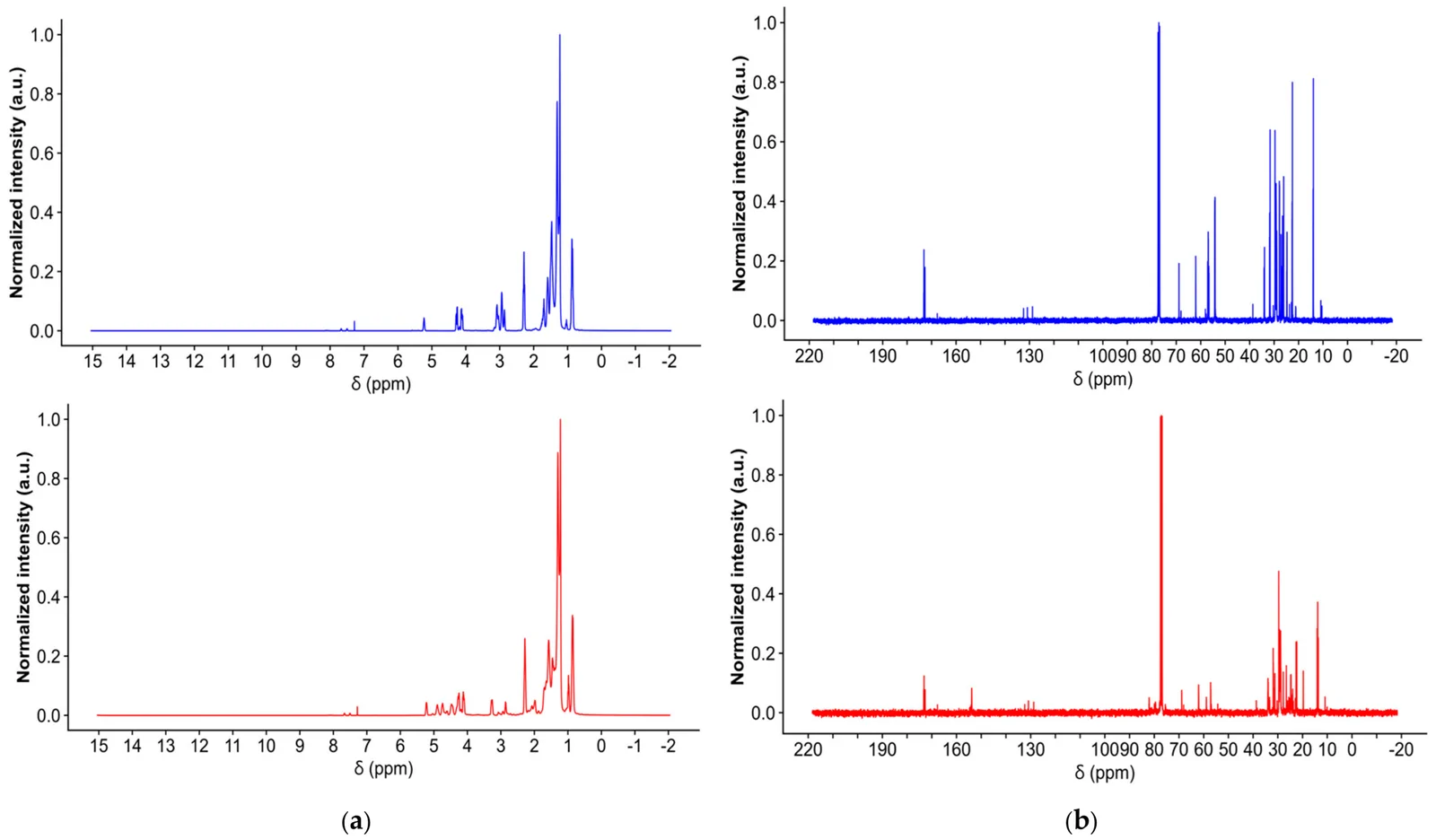
(**a**) ^1^H NMR spectra of ESBO (blue) and CSBO (red); (**b**) ^13^C NMR spectra of ESBO (blue) and CSBO (red).

According to the NMR analysis, Figure 3, the conversion of epoxidized soybean oil (ESBO) into carbonated soybean oil (CSBO) is confirmed. The ^1^H NMR spectra showed signals associated with the epoxy groups in the 2.80–3.20 ppm region. After the carbonation reaction, these signals decreased, while new resonances appeared between approximately 4.45 and 5.10 ppm in the CSBO spectrum. These changes are consistent with proton environments generated by the formation of five-membered cyclic carbonate groups [41].

The structural modification was also supported by ^13^C NMR. At approximately 155 ppm a new resonance was observed in CSBO, which is attributed to the carbonyl carbon of the cyclic carbonate functionality [41]. Concurrently, the signals around 54 to 57 ppm in ESBO, which are associated with the carbon atoms of the epoxy rings, were reduced after the reaction. These spectral changes indicate the consumption of epoxides and the formation of cyclic carbonate groups, confirming the conversion of ESBO into CSBO [41]. Moreover, the intense resonance near 77 ppm corresponds to the CDCl_3_ solvent and was not considered in the structural interpretation.

**Figure 4:**
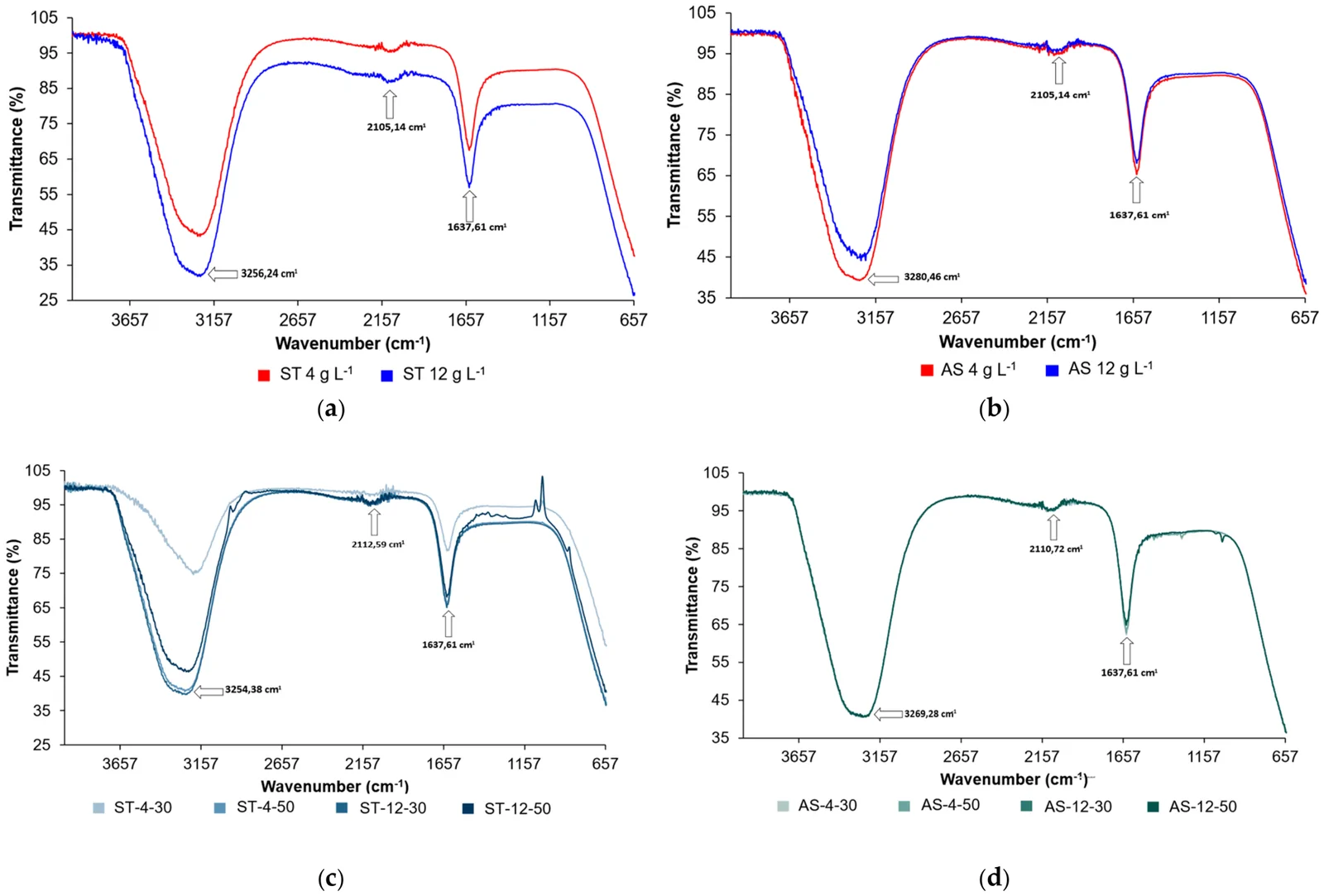
ATR-FTIR spectra of plant extracts and biologically synthesized AgNPs: (**a**) ATR-FTIR spectra of the aqueous extract of *Solanum tuberosum* (ST); (**b**) ATR-FTIR spectra of *Acca sellowiana* (AS); (**c**) ATR-FTIR spectra of ST-AgNP formulations; (**d**) ATR-FTIR spectra of AS-AgNP formulations.

Analysis of lyophilized extracts and AgNPs (Figure 4) revealed shifts in OH (3250–3270 cm^−1^), –CN (2105–2130 cm^−1^), and carbonyl (1637 cm^−1^) bands, consistent with previous reports for AgNP synthesis using *Solanum tuberosum* extract [34], indicating participation of these functional groups in nanoparticle reduction and stabilization.

### 3.4. UV-Vis Spectroscopy of AgNPs in Suspension

**Figure 5:**
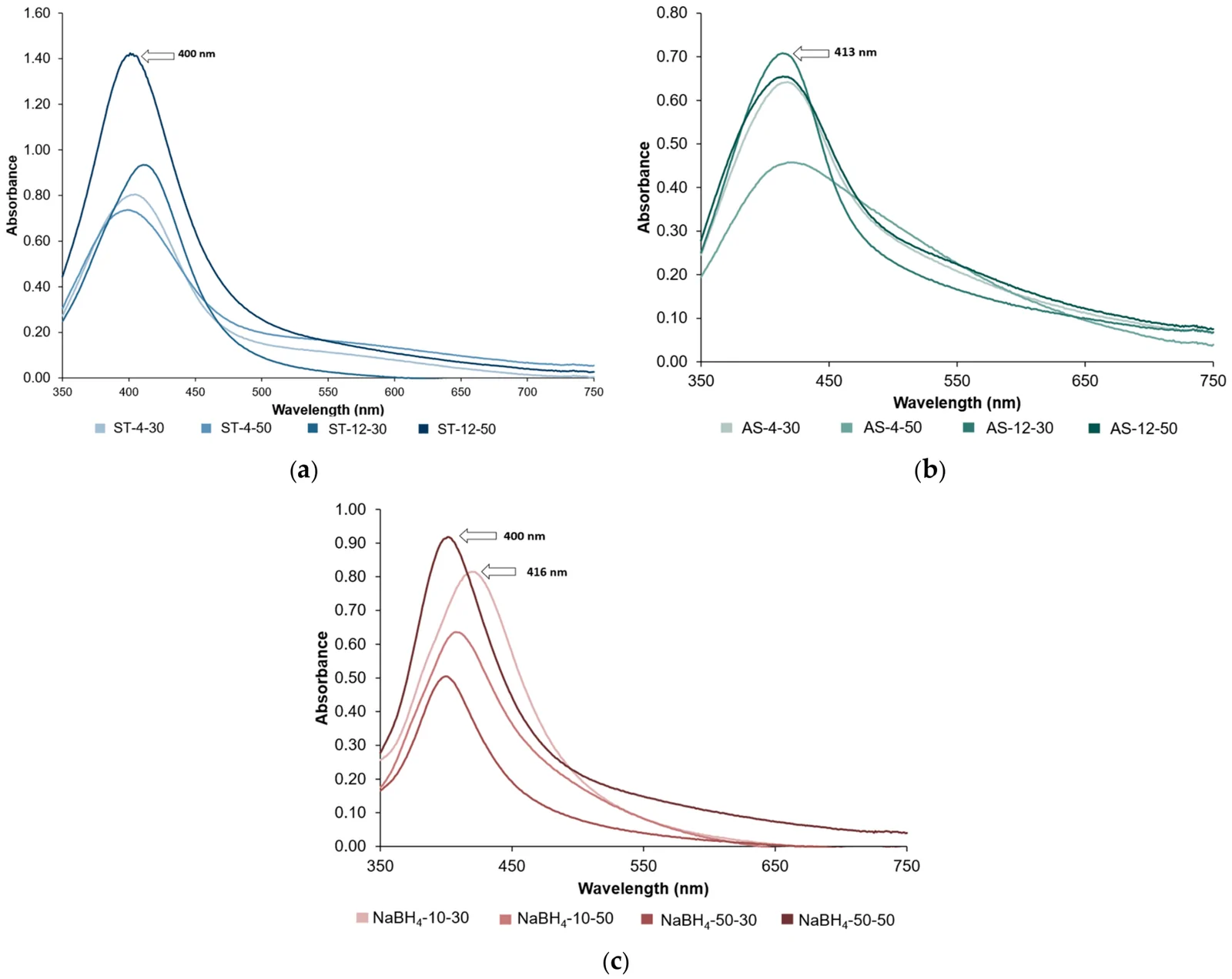
UV-Vis spectra and maximum absorbance values of the synthesized AgNPs: (**a**) *Solanum tuberosum* (ST); (**b**) *Acca sellowiana* (AS); and (**c**) NaBH_4_ formulations.

All reaction mixtures developed a dark brown coloration, which was consistent with AgNP formation through the reduction of Ag^+^ ions and generation of surface plasmon resonance (SPR) vibrations [34]. UV–Vis spectra (Figure 5a–c) showed characteristic absorption bands between 400 and 416 nm, corresponding to the SPR of silver nanoparticles, associated with AgNP formation in all synthesis systems. The presence of crystalline metallic silver was further supported by XRD results.

### 3.5. X-Ray Diffraction (XRD)

**Figure 6:**
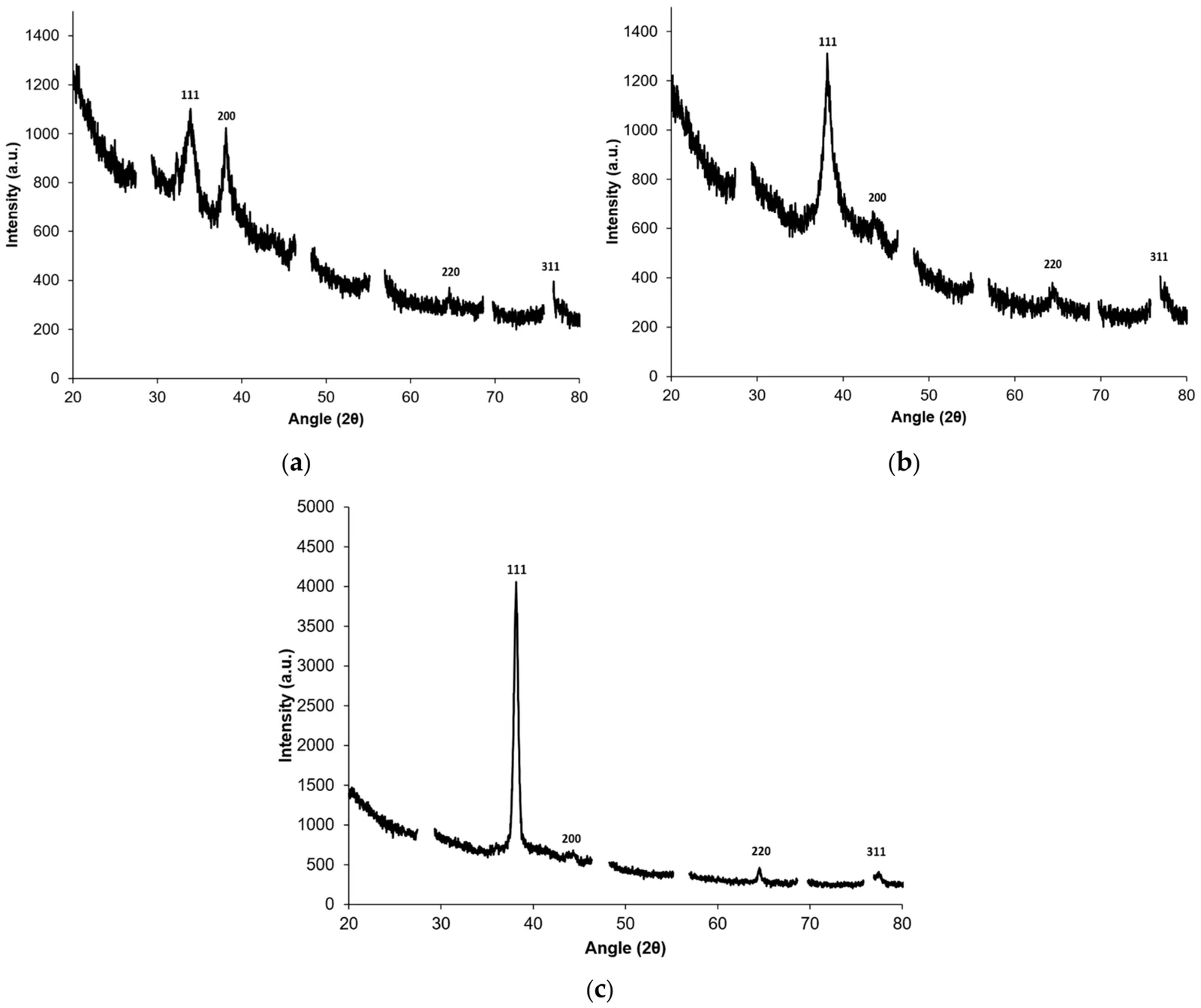
X-ray diffraction (XRD) of the synthesized AgNPs: (**a**) *Solanum tuberosum* (ST); (**b**) *Acca sellowiana* (AS); (**c**) NaBH_4_. Background signals, primarily attributed to SiO_2_, were identified and excluded from the crystallographic assignment of the sample.

XRD patterns are presented in (Figure 6a–c). A pronounced background contribution was observed due to the limited amount of recovered material and difficulties in AgNP precipitation; signals attributed to SiO_2_ were excluded from the crystallographic interpretation. Reflections associated with the (111), (200), (220), and (311) planes confirmed the face-centered cubic (FCC) structure of metallic silver nanoparticles [34]. Average crystallite sizes were determined by the Scherrer equation [34,35], values of 59.7 nm, 43.0 nm, and 33.5 nm for ST, AS, and NaBH_4_, respectively, as an approximation.

The values obtained are associated with diffracting crystalline domains rather than physical nanoparticle diameters, as illustrated by Neciosup-Puican et al. [35], who reported 9.42 nm by XRD and a hydrodynamic diameter of 21.6 nm by dynamic light scattering (DLS). The larger crystallite sizes obtained for the plant-mediated systems may be associated with the slower and more complex reduction process governed by phytochemicals acting simultaneously as reducing and capping agents, whose composition and concentration influence nucleation and subsequent crystal growth [46]. In contrast, NaBH_4_ is a strong reducing agent that promotes rapid Ag^+^ reduction and a high nucleation rate, favoring the formation of a larger number of smaller crystalline domains [46,47]. Thus, the observed size differences are consistent with the distinct reduction kinetics of the biological and chemical routes.

### 3.6. Water Contact Angle

**Figure 7:**
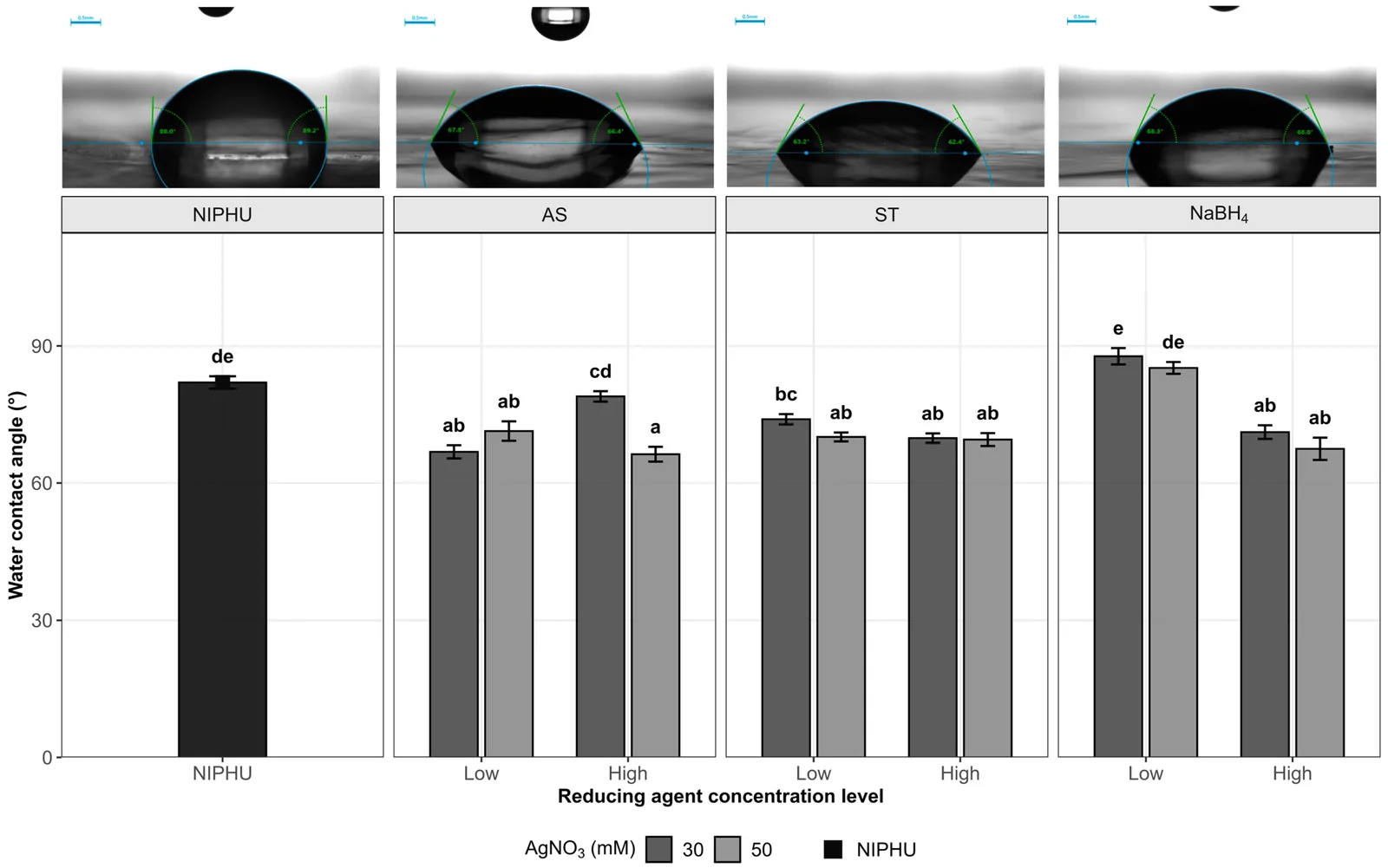
Water contact angle measurements expressed as mean ± SD (n = 12). Representative contact angle images are shown above the corresponding treatment groups; the blue scale bar in each image corresponds to 0.5 mm. For biological reducing systems, concentration levels correspond to 12 g L^−1^ (high) and 4 g L^−1^ (low) for *Acca sellowiana* (AS) and *Solanum tuberosum* (ST) extracts. For the chemical route, NaBH_4_ concentrations correspond to 50 mM (high) and 10 mM (low). Within each reducing agent level, bars represent the two AgNO_3_ concentrations evaluated (30 and 50 mM). NIPHU: corresponds to the film without AgNPs. According to one-way ANOVA and Tukey’s multiple comparison test, means not sharing the same letter differ significantly (*p* < 0.05).

All NIPHU–AgNP films exhibited lower contact angles than the unfunctionalized film (NIPHU) (Figure 7), indicating increased surface hydrophilicity following functionalization. This behavior may be associated with the presence of Ag-rich domains and changes in the organization of polar groups at the NIPHU surface and residual synthesis compounds, enhancing water interaction [48]. Similar behavior has been reported where silver incorporation decreased the contact angle, although surface roughness and particle distribution were also shown to influence final hydrophilicity [40].

Differences among treatments suggest that formulation conditions, including silver distribution, and treatment-dependent surface modifications influenced surface wettability. For example, increased hydrophilicity may be considered desirable for wound dressing applications, as it can improve cell–material interactions and facilitate antimicrobial performance [48]. Within the chemical route, NaBH_4_-10-30 and NaBH_4_-10-50 treatments exhibited higher contact angles. This behavior may reflect differences in silver reduction, retention, and surface distribution under these formulation conditions. Previous studies have shown that low concentrations of NaBH_4_ can generate fewer Ag nuclei and more sparsely distributed nanoparticles [49,50]. In PU systems, increased surface exposure of silver has also been associated with enhanced wettability [40].

Water contact angles between 40° and 90° have been reported as suitable for polymeric materials investigated for different biomedical applications, including wound dressing [12,51]. In this study, AgNP-functionalized NIPHU films showed angles between 58.86° and 73.87°; similar behavior has been reported for heparin-capped AgNP-containing polyurethane nanofibrous dressings, whose contact angles decreased and were associated with hydrophilic surfaces [5]. By contrast, soybean oil-derived NIPHU films have reported higher contact angles, from 103° to 112°, reflecting the hydrophobic contribution of long aliphatic chains [17].

### 3.7. Tensile Mechanical Test

Mechanical properties of NIPHU–AgNP films are summarized in Table 3. All treatments exhibited higher maximum stress than the unfunctionalized film (NIPHU), demonstrating that the functionalization treatments were associated with increased mechanical resistance, probably from multiple formulation-dependent factors, including Ag-rich domain distributions and interactions with functional groups in the NIPHU matrix, which may contribute to differences in stress transfer during deformation [48] while preserving their deformability. Given that soybean oil-derived NIPHUs typically exhibit tensile strengths between 0.5 and 1.13 MPa [41], all functionalized formulations showed a substantial increase in mechanical resistance. For comparison, tensile strengths of approximately 2.5 to 16 MPa and elongations above 70% have been proposed for flexible polymeric systems in which mechanical compliance and the ability to accommodate large deformations are required, including wound dressing materials [52].

In previous studies, Gholami and Yeganeh [53] developed soybean oil-derived NIPU membranes with a maximum stress of approximately 6 MPa and elongations close to 200%, demonstrating that bio-based NIPU networks can combine moderate strength and high flexibility. Likewise, other studies reported 7.13 MPa and 122.34% elongation for pristine PU nanofibers, whereas the incorporation of 1% heparin-capped AgNPs increased the tensile strength to 11.9 MPa and elongation to 239% [5]

The highest maximum stresses were obtained for NaBH_4_-10-50 (13.12 ± 1.87 MPa), ST-4-30 (12.87 ± 1.26 MPa), and NaBH_4_-50-30 (12.78 ± 0.72 MPa), whereas other formulations show more moderate reinforcement. In comparison with the unfunctionalized NIPHU, this behavior may be associated with the establishment of interfacial interactions between the polymeric matrix and the Ag-containing domains. Favorable filler–matrix interactions can promote stress transfer across the interface through intermolecular interactions, whereas particle agglomeration and interfacial defects reduce the effective contact area and may generate local stress concentration sites [54]. Similar trends have been observed in AgNP-containing polymer systems, where low or intermediate nanoparticle loadings increase stress transfer, whereas excessive loading can promote agglomeration and local stress concentration [5,55]. Moreover, silver has also been reported to interact with polar N- and O-containing groups of the polymer backbone, which may alter the hydrogen bonding and restrict chain mobility [40,56].

Among biologically synthesized formulations, ST-4-30 exhibited the most favorable balance between strength and deformability. Its response may reflect a favorable combination of silver incorporation, surface distribution, and interactions introduced by the *Solanum tuberosum* mediated process [34,48]. Its toughness (4.13 ± 0.32 MJ m^−3^) indicates a greater capacity to absorb mechanical energy before failure than the unfunctionalized NIPHU (1.11 ± 0.43 MJ m^−3^). This combination is particularly relevant for polymeric systems that must withstand handling-induced stresses while remaining deformable to conform to moving or irregular surfaces [57,58]. Films synthesized with *Acca sellowiana* extract generally showed lower mechanical performance, likely due to differences in extract composition, reduction efficiency, and nanoparticle–matrix interactions.

Moreover, the increase in maximum stress was not accompanied by an increase in stiffness. The NaBH_4_-10-50 treatment showed a statistically significant difference in secant modulus at 10% in comparison with the unfunctionalized film. This behavior may be associated with the specific conditions generated during chemical functionalization, nanoparticle distribution, and related to the smaller crystallite size of NaBH_4_-derived AgNPs (33.5 nm), yielding a larger interfacial area and more efficient stress transfer within the polymer network [48], consistent with observations in polyurethane systems where mechanical response depends on nanoparticle dispersion and interfacial adhesion [48].

For comparison with previous studies, tensile modulus values close to 1.5 to 3 MPa are reported for natural skin and have been used as mechanical reference in elastomeric polyurethane systems, including potential wound dressing applications [59]. Although the two quantities are not interchangeable, they provide information on resistance to deformation and allow a qualitative assessment of film stiffness. Previous studies have emphasized that matching the stiffness of the dressing to that of skin can improve conformability and reduce mechanical mismatch at the material–tissue interface [58,59]. Additionally, PUs with AgNPs have shown that nanoparticle incorporation can modify Young’s modulus depending on its dispersion and concentration [5].

### 3.8. Thermal Characterization

**Figure 8:**
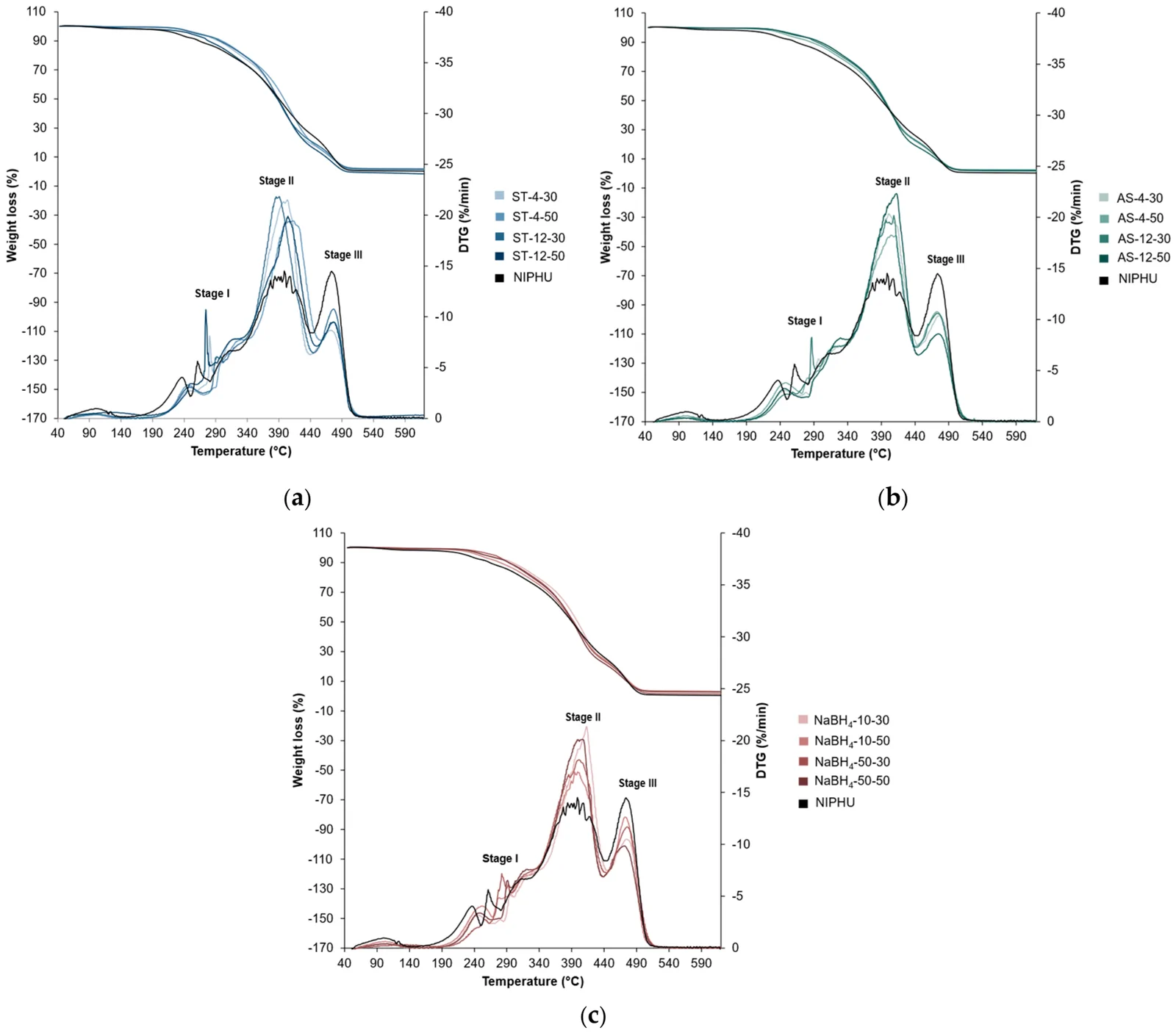
Thermograms of the synthesized NIPHU-AgNPs: derivative thermogravimetric curve (DTG) and thermogravimetric curve (TGA) of (**a**) *Solanum tuberosum* (ST); (**b**) *Acca sellowiana* (AS); and (**c**) NaBH_4_ formulations. NIPHU: corresponds to the film without AgNPs.

TGA/DTG curves of NIPHU–AgNP films are presented in Figure 8. The functionalized films showed slight variations in degradation intensity and peak temperatures between 350 and 450 °C, attributed to differences in nanoparticle–polymer interactions, network heterogeneity, and the presence of residual components associated with the different reduction routes. Similar effects have been reported in polyurethane composites with inorganic fillers such as AgNPs [48]. Despite these variations, all formulations remained thermally stable up to approximately 200 °C, consistent with previous reports for soybean oil-derived NIPHUs [41]. These results indicate that the functionalization treatment did not compromise the initial thermal stability of the NIPHU matrix. From an application perspective, no universal decomposition temperature threshold has been established, for example, as a wound dressing material. Nevertheless, the material should remain thermally stable above physiological temperatures.

**Figure 9:**
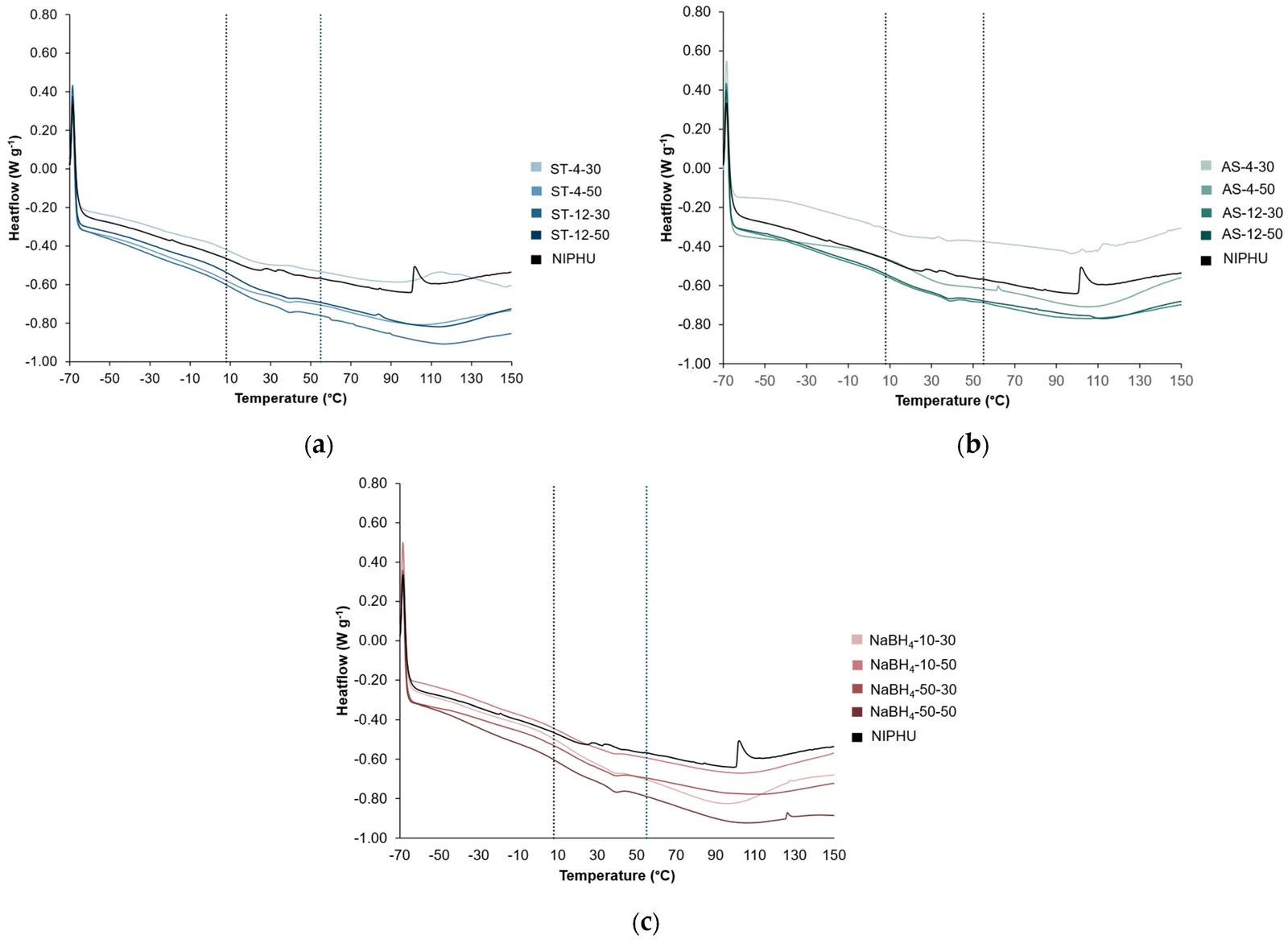
DSC curves of (**a**) *Solanum tuberosum* (ST); (**b**) *Acca sellowiana* (AS); and (**c**) NaBH_4_ formulations. NIPHU: corresponds to the film without AgNPs. One DSC measurement was performed for each formulation (n = 1).

Differential scanning calorimetry (DSC) curves in Figure 9 showed formulation-dependent variations in the glass transition temperature (T_g_) of the AgNP-functionalized NIPHU films, with individual measured values ranging from 9.89 °C to 59.82 °C. Several evaluated formulations, including ST-4-50 (36.48 °C), AS-12-50 (34.87 °C), AS-4-30 (31.93 °C), AS-12-30 (36.53 °C), and NaBH_4_-50-50 (36.49 °C), showed T_g_ values within a relatively narrow intermediate range, whereas the remaining formulations exhibited lower or higher values. Previous studies on soybean oil-derived NIPHUs reported that the presence of different urethane- and amide-containing domains has been associated with distinct segmental mobilities and multiple thermal transitions [17]. In addition, the cycloaliphatic CHM incorporated into the NIPHU matrix has previously been related to restricted chain mobility and increased T_g_ [18,41].

Silver nanoparticles have been reported to restrict molecular motion in polyurethane matrices, associated with an increase in T_g_ when the particles are well dispersed [40]. This behavior is consistent with the formation of polymer–nanoparticle interfacial regions that limit segmental mobility. However, the effect of nanoparticle incorporation on T_g_ depends on nanoparticle dispersion, polymer–particle interactions, phase organization, and changes in hydrogen bonding. Previous studies showed that AgNPs can hinder hydrogen bond formation and restrict soft segment molecular motions [60], whereas Paul et al. [40] reported that microscale phase separation may reduce T_g_ despite silver incorporation. In NIPHU systems, Raftopoulos et al. [18] further demonstrated that T_g_ is highly sensitive to chain packing, free volume, hydrogen bonding, and segmental mobility.

Elastomeric materials generally exhibit greater compliance above T_g_, whereas operation below this temperature is associated with a more rigid response. Therefore, a glass transition temperature close to the intended service temperature is important for evaluating flexibility and segmental mobility of polymeric films; for example, for flexible wound dressing applications, T_g_ should be considered relative to the intended service temperature, including physiological temperature [61]. This interpretation needs to be considered in conjunction with tensile results, since the actual flexibility depends on both thermal transitions and mechanical response of the complete polymer network.

### 3.9. PBS 1X and Water Absorption

Maximum fluid absorption results are shown in Table 4. Particularly in PBS these values ranged from 4.72 ± 0.72% for NaBH_4_-10-30 to 15.36 ± 8.56% for ST-4-50; however, no significant differences were observed among the functionalized films and the unfunctionalized NIPHU (12.82 ± 1.90%). These results indicate that functionalization did not systematically modify the maximum PBS uptake capacity of the NIPHU matrix.

Regarding water absorption, the unfunctionalized NIPHU exhibited a maximum absorption of 4.79 ± 0.71%, whereas ST-12-50 and ST-4-30 were significantly higher. The remaining formulations were statistically comparable with the NIPHU without AgNPs. Therefore, the increase in water uptake should not be attributed to AgNP incorporation alone; rather, it appears to depend on the specific functionalization conditions and the resulting changes in polymer surface and polymer additive interactions.

Water uptake in NIPHU networks is governed by a balance between the hydrophilic hydroxyl and urethane groups generated during cyclic carbonate aminolysis and the constraints imposed by crosslink density and hydrophobic polymer segments. Silver-containing domains and residual compounds introduced may alter hydrogen bonding interactions, and accessibility of polar groups to water from the reducing system. Previous studies on NIPHU networks have demonstrated that swelling behavior can differ between distilled water and PBS and depends on network chemistry and incorporated nanostructures [62].

The moderate fluid uptake observed should be interpreted in relation to the architecture of the films and intended application. Dense PU films generally exhibit lower fluid absorption than porous foams or hydrogel dressings. Previous studies in crosslinked soybean oil-based waterborne PU films reported absorption approximately 3 wt% after 24 h in water [63], whereas highly hydrophilic NIPU hydrogel networks can absorb from 200 wt% in water up to 215 wt% in PBS [62]. For comparison, semi-permeable polyurethane film dressings are intended for superficial or epithelializing wounds with low exudate; therefore, the moderate fluid uptake observed may be appropriate for a flexible film in low-exudate wounds or as a wound contact layer [64,65].

### 3.10. Total Silver Content

**Figure 10:**
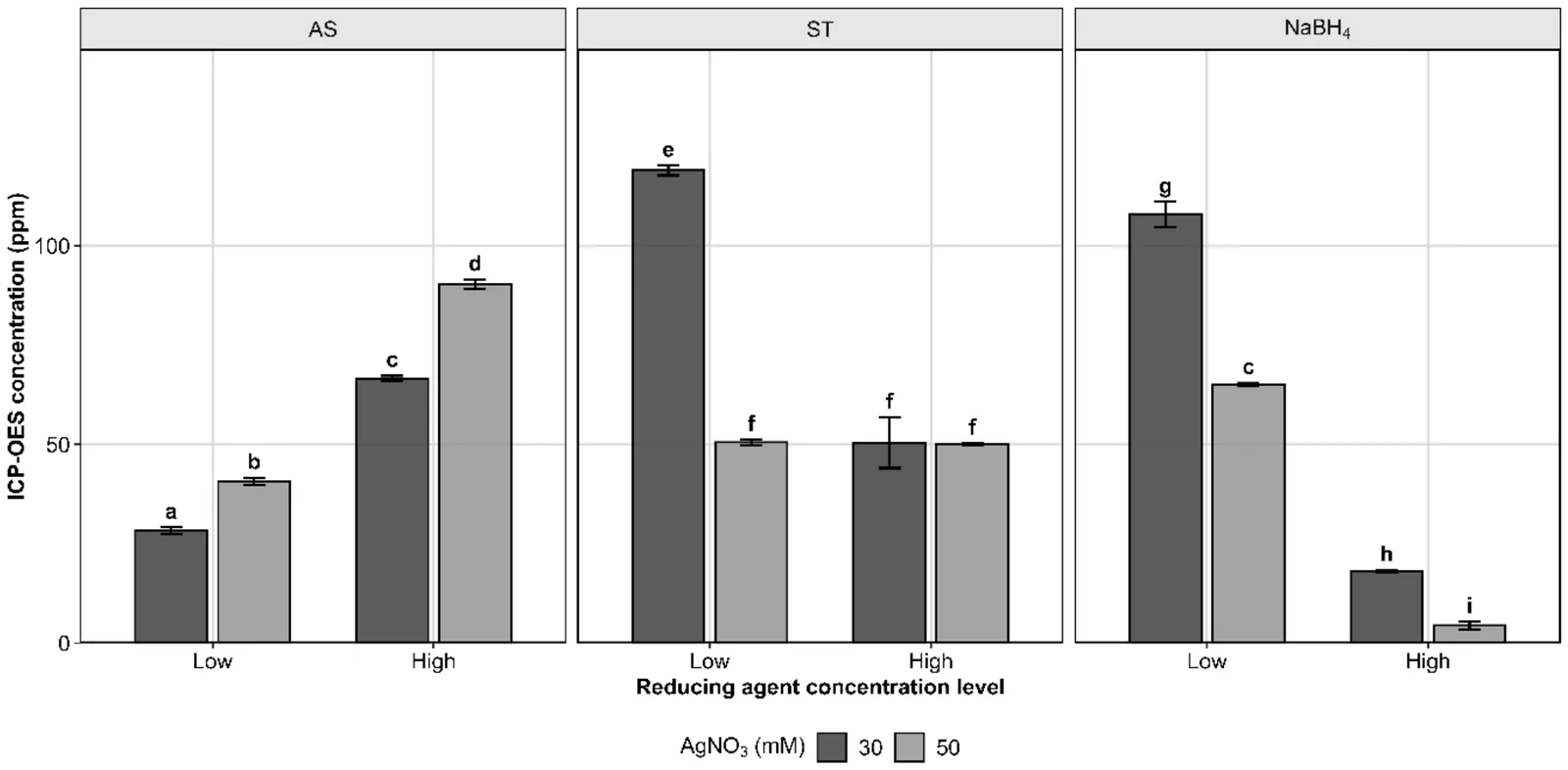
Total silver content measurements by ICP-OES expressed as mean ± SD (n = 3). For biological reducing systems, concentration levels correspond to 12 g L^−1^ (high) and 4 g L^−1^ (low) for *Acca sellowiana* (AS) and *Solanum tuberosum* (ST) extracts. For the chemical route, NaBH_4_ concentrations correspond to 50 mM (high) and 10 mM (low). Within each reducing agent level, bars represent the two AgNO_3_ concentrations evaluated (30 and 50 mM). Statistical differences among treatments were evaluated using factorial permutation ANOVA, followed by a studentized permutation max-T post hoc test with family-wise error rate (FWER) control, means not sharing the same letter differ significantly (*p* < 0.05).

Silver incorporation was observed to be strongly dependent on the reducing system and formulation conditions, see Figure 10. Among biological routes, ST-4-30 exhibited the highest silver content (119.01 ± 1.24 ppm), whereas the other ST formulations did not present a statistical difference. In contrast, AS formulations showed a progressive increase in silver incorporation by increasing AgNO_3_ concentrations, reaching 90.29 ± 1.22 ppm for AS-12-50. Regarding the chemical route, lower concentrations of NaBH_4_ showed higher values for NaBH_4_-10-30 (107.89 ± 3.21 ppm), whereas by increasing NaBH_4_ concentrations silver incorporation decreased, reaching 4.44 ± 0.95 ppm for NaBH_4_-50-50. Therefore, silver incorporation appears not to be controlled only by the availability of Ag^+^ but by the balance between silver ion reduction, nucleation and growth, stabilization, and subsequent retention within the polymer matrix.

According to the lyophilized extract characterization, AS showed a substantially higher phenolic content, reducing sugars, and antioxidant activity than ST, indicating a greater concentration of compounds capable of acting as reducing and capping agents. Nevertheless, ST-4-30 reached higher values than any AS formulation, demonstrating that higher reducing capacity does not increase silver incorporation. Increasing the concentration of phytochemicals can initially accelerate the reduction and nucleation, whereas excessive capping and reducing compounds may promote rapid particle formation, aggregation, polydispersity or over-stabilization [46]. A recent study with *Solanum tuberosum* extract showed that, by increasing the extract concentration, AgNPs reached an optimum, while further increases broadened the SPR band and promoted polydispersity and aggregation [66]. Therefore, ST-4-30 retention may represent a favorable balance between reduction rate and nanoparticle association.

Moreover, chemical route values suggest that each reducing system produces different nucleation and growth regimes. X-ray diffraction for NaBH_4_ treatments showed smaller crystallites, compatible with the rapid nucleation expected for strong chemical reducing agents. This chemical route has been reported to involve extremely rapid formation of silver species followed by colloidal growth and aggregation processes [67]. At high NaBH_4_ concentrations, a greater fraction of silver may therefore have nucleated in the liquid phase rather than being associated with the films and subsequently been removed during washing. This mechanism would be consistent with marked reduction in the silver content at the highest NaBH_4_ level. However, total silver content did not parallel with the mechanical response and may be associated with the effective polymer–silver interfacial area, well-dispersed nanoscale domains provide a larger interfacial area for polymer–particle interactions, whereas aggregation reduces the specific contact area and may create heterogeneous or defect-rich regions [54,56]. ST-4-30 combined the highest silver content with the most favorable mechanical performance. In contrast, NaBH_4_-10-50 and NaBH_4_-50-30 also exhibited high tensile strengths despite their markedly different silver contents. Therefore, the mechanical response was not directly proportional to total silver content, indicating that silver loading alone cannot explain the observed mechanical behavior.

The silver content measured in the NIPHU-AgNP films can be contextualized by comparison with commercially available silver-containing polymeric and fibrous materials. For example, Rigo et al. [68] characterized four commercial dressing used in burn care (ACTICOAT^TM^ Flex 3, ACTISORB^®^ Silver 220, AQUACEL^®^ Ag, and Mepilex^®^ Ag); despite all products being clinically intended as silver dressings, their total silver loading varied markedly, from approximately 0.03 to 1.39 mg Ag cm^−2^, and their release behavior depended on the surrounding medium. A similar lack of proportionality between total silver content and silver availability was demonstrated in the literature by comparing AQUACEL Ag+ Extra, ACTICOAT, Silvercel Hydro-Alginate, and lalugen Plus. The dressings contained approximately 120, 919, 864, and 120 µg Ag cm^−2^, respectively; however, a subsequent extraction into a protein-containing culture medium showed that lalugen Plus released the highest silver concentration despite having one of the lowest total silver loadings, whereas Silvercel contained more silver but released the lower concentration [69]. Therefore, these findings demonstrate that dressing architecture, silver speciation, and interactions between silver and polymeric or fibrous matrices can be as important as silver loading when determining silver bioavailability [69].

Khansa et al., in a comprehensive review of silver-based wound care, reported that approximately 30 to 40 ppm Ag^+^ is required for effective bactericidal activity, whereas cytotoxic effects toward keratinocytes and fibroblasts have been observed from approximately 33 ppm in monolayer cultures and around 60 ppm in a three-dimensional collagen model [70]. On this basis, it is proposed that an ideal silver dressing should sustain a therapeutic silver concentration of at least 30 ppm while remaining below levels associated with local toxicity (around 60 ppm) [70]. Therefore, future silver release studies should determine whether the AgNPs incorporated into the NIPHU can provide sustained concentrations within an antimicrobial cytocompatibility window.

### 3.11. Scanning Electron Microscopy (SEM)

**Figure 11:**
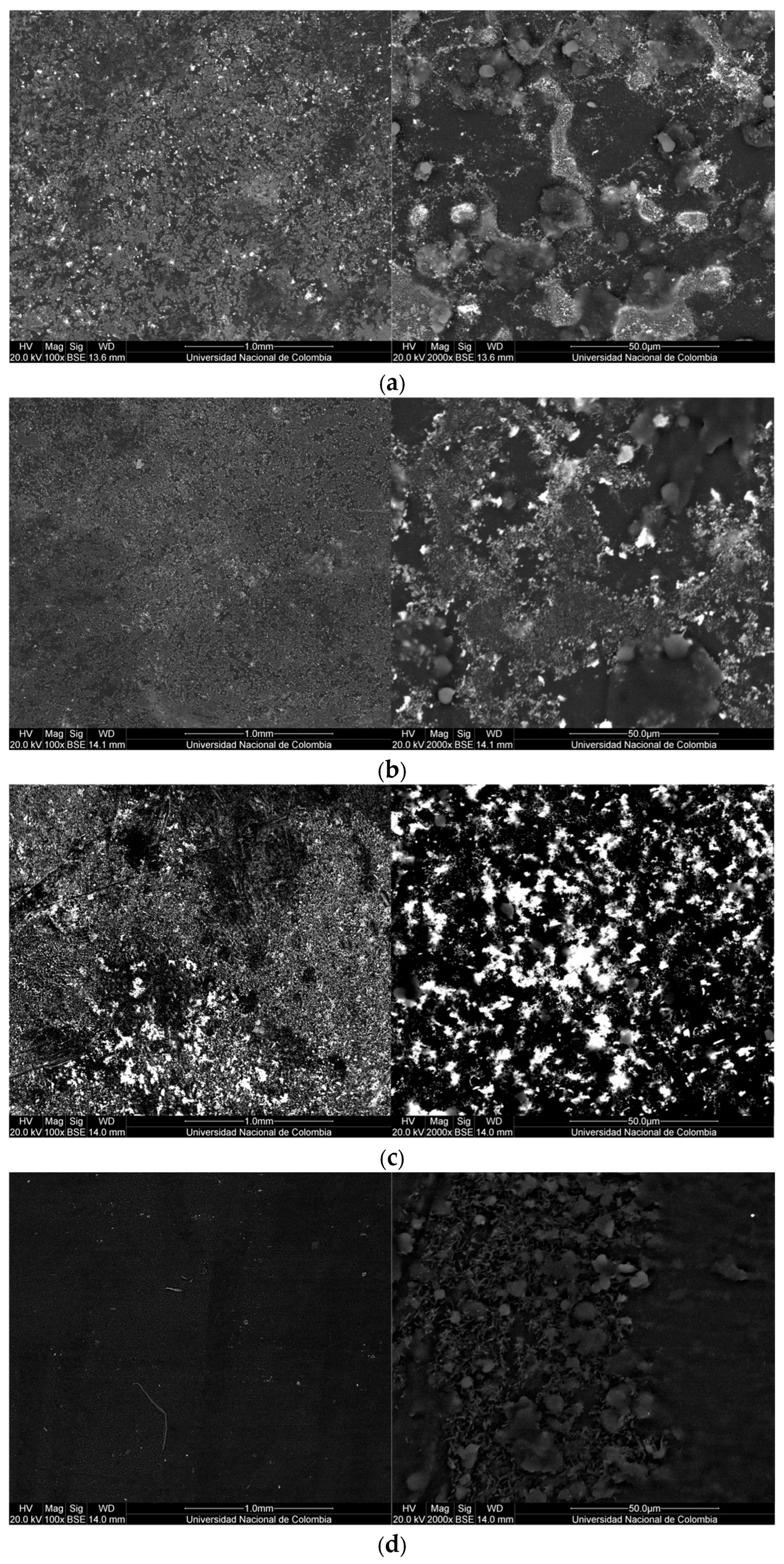
SEM-BSE micrographs of AgNP-functionalized and -unfunctionalized NIPHU films. (**a**) *Solanum tuberosum*-mediated AgNP synthesis (ST-4-30); (**b**) *Acca sellowiana*-mediated AgNP synthesis (AS-12-50); (**c**) NaBH_4_-mediated AgNP synthesis (NaBH_4_-50-30); and (**d**) unfunctionalized NIPHU. For each micrograph, the image on the left corresponds to 100× magnification and the image on the right to 2000× magnification.

Backscattered electron (BSE) SEM analysis revealed differences in the surface morphology and distribution of Ag-rich domains among the treatments evaluated (Figure 11). The unfunctionalized NIPHU film (Figure 11d) exhibited a predominantly dark and comparatively homogeneous surface, whereas functionalized films showed bright features distributed over the polymer matrix. In BSE imaging, elements with higher atomic numbers (Z) generate a stronger BSE signal and consequently appear brighter than low-Z elements. Considering the atomic number of silver (Z = 47) relative to the light elements constituting the polymeric matrix, the bright regions may be associated with Ag-rich domains, while the darker background corresponds predominantly to the organic polymer phase [71]. However, since the samples were sputter-coated prior to SEM analysis, the contributions of high-Z elements to the BSE contrast cannot be completely excluded. As identical coating conditions were applied to all samples, differences in the bright features were interpreted as surface heterogeneity potentially associated with Ag domains and were considered together with the ICP-OES results. Moreover, the unfunctionalized NIPHU was subjected to the same coating procedure but did not exhibit the localized bright clusters observed in the functionalized films, supporting that these features are unlikely to arise solely from the coating.

Similar BSE contrast has been reported in AgNP-containing thermoplastic polyurethane, where the polymeric matrix appeared as a dark background and bright nanoscale features were assigned to AgNPs [72]. Likewise, Hengsbach et al. reported that metallic nanoparticles appeared as bright regions against an organic polymer background in BSE images of polymeric nanofibers, while a complementary elemental analysis was used to support their findings [73].

The biologically functionalized films, AS-12-50 using *Acca sellowiana* (Figure 11b) and ST-4-30 using *Solanum tuberosum* (Figure 11a), exhibited bright regions distributed over the NIPHU surface, together with evident clusters, particularly at higher magnifications. Although both treatments showed route-specific surface patterns, their morphology revealed treatment-dependent surface heterogeneity, while silver incorporation was supported by ICP-OES results. Plant-mediated AgNP synthesis involves phytochemical compounds acting simultaneously as reducing and stabilizing agents, and differences in extract composition, pH, concentration, temperature, and reaction conditions can affect nucleation, growth, particle size, and stability [74]. In the present study, this interpretation is consistent with the ICP-OES results, since AS-12-50 and ST-4-30 exhibited higher total silver contents than the NaBH_4_-50-30 treatment, although the SEM-BSE images should be interpreted qualitatively as evidence of Ag-rich surface domains rather than as a direct measurement of total silver concentration.

Nevertheless, complementary techniques could provide additional information on nanoparticle distribution and surface characteristics. SEM-EDS has been successfully used in silver-containing polymer nanocomposites to confirm the elemental presence of silver and evaluate its distribution within polymer films [75]. In parallel, atomic force microscopy (AFM) has been applied to evaluate morphological changes in polymer matrices [76]. Therefore, the combined use of SEM-EDS and AFM should be considered in future studies to complement SEM-BSE observations and provide a more detailed assessment of distribution and surface organization in functionalized films.

In contrast, the NaBH_4_-functionalized film, NaBH_4_-50-30, (Figure 11c) also showed prominent bright regions, although its spatial distribution differs in comparison with AS-12-50 and ST-4-30. NaBH_4_ is a strong reducing agent that promotes the rapid reduction of silver and favors high nucleation rates, which can lead to the formation of numerous small nanoparticles [47]. Similar behavior has been described for strong reducing conditions, where faster reduction rates favor smaller particle sizes, whereas slower reduction may promote particle growth or agglomeration [71,74]. The apparent abundance of bright domains in Figure 11 is also consistent with the ICP-OES results, where the chemical formulations with a high concentration of the reducing agent showed the lowest total silver concentrations.

The localized heterogeneity observed may be associated with non-uniform AgNP deposition or aggregation. AgNP formation and stability are influenced by synthesis parameters such as reducing agent and silver nitrate concentrations, pH, reduction temperature, time, and mixing conditions [46,47]. In plant-mediated systems, excessive extract concentration may alter nucleation and stabilization, promoting polydispersity and aggregation [66]. Likewise, temperature can modify particle growth and aggregation during NaBH_4_ reduction by altering reaction kinetics and colloidal interactions [67]. Therefore, future optimization of the NIPHU functionalization process could focus on controlling these parameters.

## 4. Conclusions

Soybean oil-derived NIPHU films were successfully functionalized with silver nanoparticles obtained through chemical reduction with NaBH_4_ and biological reduction using *Solanum tuberosum* and *Acca sellowiana* peel extracts. The functionalized films exhibited route-dependent differences in silver incorporation, surface morphology, wettability, mechanical response, thermal behavior, and fluid interaction. The biological reduction route using plant extracts therefore represents a feasible alternative to conventional methods while enabling the valorization of agro-industrial residues. Among the biological treatments evaluated, ST-4-30 showed the most balanced overall mechanical and physicochemical performance. The combination of moderate wettability, high mechanical resistance and deformability, thermal stability, and fluid interaction supports further investigation for potential biomedical applications. However, changes in material properties should not be attributed solely to AgNP incorporation but rather to the influence of the reducing system, nanoparticle distribution, formulation conditions, and interactions between Ag-rich domains and the polymeric matrix. Further studies should address silver release, antimicrobial activity, cytocompatibility, and degradation behavior to better understand the performance of these functionalized materials.

## Figures and Tables

**Table 1:** Reducing agents and concentrations evaluated.

Reducing Agent	Concentration of Reducing Agent	Concentration AgNO_3_
*Acca sellowiana* (AS)	4 g L^−1^	30 mM
*Acca sellowiana* (AS)	4 g L^−1^	50 mM
*Acca sellowiana* (AS)	12 g L^−1^	30 mM
*Acca sellowiana* (AS)	12 g L^−1^	50 mM
*Solanum tuberosum* (ST)	4 g L^−1^	30 mM
*Solanum tuberosum* (ST)	4 g L^−1^	50 mM
*Solanum tuberosum* (ST)	12 g L^−1^	30 mM
*Solanum tuberosum* (ST)	12 g L^−1^	50 mM
NaBH_4_	10 mM	50 mM
NaBH_4_	10 mM	30 mM
NaBH_4_	50 mM	50 mM
NaBH_4_	50 mM	30 mM

AS: *Acca sellowiana*; ST: *Solanum tuberosum*; NaBH_4_: sodium borohydride, AgNO_3_: silver nitrate. For biological routes, reducing agent concentration refers to the concentration of lyophilized extract expressed in g L^−1^, whereas for the chemical route it refers to the molar concentration of NaBH_4_. Each reducing agent concentration was evaluated in combination with 30 or 50 mM AgNO_3_. For NaBH_4_, a molar ratio of Ag^+^ to BH_4_^−^ greater than 1:1 was ensured, guaranteeing a silver excess in the reaction.

**Table 2:** Summary of plant extract characterization results.

Extract	Total Phenolic Content (mg GAE g^−1^ Sample)	Reducing Sugars (mg Glucose eq g^−1^ Sample)	Antioxidant Activity (µmol TROLOX eq g^−1^ Sample)
*Acca sellowiana* (AS)	57.97 ± 2.78 ^a^	221.85 ± 14.48 ^a^	94.83 ± 2.22 ^a^
*Solanum tuberosum* (ST)	3.75 ± 0.16 ^b^	17.05 ± 3.65 ^b^	24.07 ± 0.95 ^b^

AS: *Acca sellowiana*; ST: *Solanum tuberosum*; GAE: gallic acid equivalent; eq: equivalent. Results are expressed as mean ± SD (n = 3). Within each parameter, values not sharing the same superscript letter differ significantly according to one-way ANOVA followed by Tukey’s post hoc test (*p* < 0.05).

**Table 3:** Tensile mechanical test results for NIPHU-AgNP films.

Sample	Thickness (mm)	Maximum Stress (MPa)	Maximum Elongation (%)	Secant Modulus (MPa)	Toughness (MJ m^−3^)
AS-12-30	0.17 ± 0.01	4.15 ± 0.70 ^b^	92.40 ± 7.64 ^a^	2.16 ± 0.13 ^ab^	1.44 ± 0.22 ^ab^
AS-12-50	0.28 ± 0.03	6.90 ± 0.97 ^bcdef^	122.75 ± 10.87 ^ab^	1.74 ± 0.52 ^a^	2.4 ± 0.22 ^bc^
AS-4-30	0.22 ± 0.01	4.78 ± 0.93 ^bc^	90.50 ± 8.50 ^a^	2.44 ± 0.29 ^ab^	1.54 ± 0.32 ^ab^
AS-4-50	0.29 ± 0.04	8.51 ± 1.30 ^ef^	119.63 ± 14.69 ^ab^	2.51 ± 0.74 ^ab^	2.86 ± 0.54 ^cd^
ST-12-30	0.25 ± 0.03	7.87 ± 0.78 ^def^	111.43 ± 5.71 ^ab^	3.01 ± 0.12 ^abc^	2.64 ± 0.29 ^bc^
ST-12-50	0.24 ± 0.01	5.52 ± 1.10 ^bcd^	105.01 ± 8.23 ^ab^	2.62 ± 0.01 ^ab^	2.06 ± 0.48 ^bc^
ST-4-30	0.32 ± 0.02	12.87 ± 1.26 ^g^	131.27 ± 8.77 ^b^	2.85 ± 0.12 ^ab^	4.13 ± 0.32 ^e^
ST-4-50	0.25 ± 0.04	5.59 ± 0.81 ^bcde^	105.07 ± 7.17 ^ab^	2.41 ± 0.63 ^ab^	1.94 ± 0.35 ^abc^
NaBH_4_-10-30	0.28 ± 0.09	7.59 ± 1.58 ^cdef^	118.06 ± 11.51 ^ab^	2.19 ± 0.67 ^ab^	2.44 ± 0.55 ^bc^
NaBH_4_-10-50	0.25 ± 0.05	13.12 ± 1.87 ^g^	112.09 ± 4.68 ^ab^	5.87 ± 1.59 ^c^	4.22 ± 0.63 ^e^
NaBH_4_-50-30	0.27 ± 0.02	12.78 ± 0.72 ^g^	123.70 ± 4.15 ^ab^	3.53 ± 0.85 ^bc^	3.99 ± 0.14 ^de^
NaBH_4_-50-50	0.32 ± 0.01	9.38 ± 1.91 ^f^	119.30 ± 5.69 ^ab^	2.99 ± 0.14 ^abc^	3.11 ± 0.61 ^cde^
NIPHU	0.27 ± 0.05	1.80 ± 0.49 ^a^	107.16 ± 18.65 ^ab^	2.45 ± 0.71 ^ab^	1.11 ± 0.43 ^a^

Results are expressed as mean ± SD (n = 3 from independent films); for the NIPHU, N = 5 independent films, with three technical measurements per film. Secant modulus was calculated at 10% strain. Toughness was calculated as the area under the stress–strain curve up to fracture using the trapezoidal rule and is reported in MJ m^−3^. For statistical analysis, secant modulus data were log-transformed to meet ANOVA assumptions; original values are reported. Means not sharing the same letter differ significantly according to ANOVA and Tukey’s test (*p* < 0.05).

**Table 4:** Absorption results for NIPHU-AgNP films.

Sample	PBS Maximum Absorption (wt%)	Water Maximum Absorption (wt%)
AS-12-30	8.83 ± 5.80 ^a^	9.83 ± 0.48 ^abc^
AS-12-50	5.56 ± 1.40 ^a^	7.81 ± 0.51 ^abc^
AS-4-30	8.02 ± 1.34 ^a^	5.77 ± 1.18 ^abc^
AS-4-50	6.70 ± 3.67 ^a^	12.21 ± 4.15 ^abc^
ST-12-30	7.44 ± 0.45 ^a^	6.70 ± 2.33 ^abc^
ST-12-50	9.91 ± 4.04 ^a^	13.71 ± 3.47 ^c^
ST-4-30	8.52 ± 4.08 ^a^	13.05 ± 6.45b ^c^
ST-4-50	15.36 ± 8.56 ^a^	5.11 ± 0.35 ^ab^
NaBH_4_-10-30	4.72 ± 0.72 ^a^	10.48 ± 3.34 ^abc^
NaBH_4_-10-50	13.04 ± 2.88 ^a^	7.97 ± 2.31 ^abc^
NaBH_4_-50-30	7.91 ± 2.26 ^a^	8.19 ± 1.90 ^abc^
NaBH_4_-50-50	5.26 ± 1.92 ^a^	7.88 ± 0.21 ^abc^
NIPHU	12.82 ± 1.90 ^a^	4.79 ± 0.71 ^a^

Results are expressed as mean ± SD (n = 3). Means not sharing the same letter differ significantly according to ANOVA and Tukey’s test (*p* < 0.05).

## Data Availability

The original contributions presented in this study are included in the article. Further inquiries can be directed to the corresponding author.

## Abbreviations

The following abbreviations are used in this manuscript:

AgNPs	Silver nanoparticles
AgNO_3_	Silver nitrate
AFM	Atomic force microscopy
ANOVA	Analysis of variance
AS	*Acca sellowiana*
ASTM	ASTM International
ATR-FTIR	Attenuated total reflectance Fourier transform infrared spectroscopy
Au/Pd	Gold/palladium
BDA	1,4-butanediamine
BSE	Backscattered electron
CHM	1,3-cyclohexanebis(methylamine)
CSBO	Carbonated soybean oil
DLS	Dynamic light scattering
DMA	Dynamic mechanical analysis
DSC	Differential scanning calorimetry
DTG	Derivative thermogravimetry
EDS	Energy-dispersive X-ray spectroscopy
ESBO	Epoxidized soybean oil
FCC	Face-centered cubic
FWER	Family-wise error rate
GAE	Gallic acid equivalents
HV	High voltage
ICP-OES	Inductively coupled plasma optical emission spectrometry
ISO	International Organization for Standardization
NaBH_4_	Sodium borohydride
NIPHU	Non-isocyanate polyhydroxyurethane
NIPHU-AgNPs	Non-isocyanate polyhydroxyurethane with silver nanoparticles
NIPU	Non-isocyanate polyurethane
NMR	Nuclear magnetic resonance
PBS	Phosphate-buffered saline
PHU	Polyhydroxyurethane
PU	Polyurethane
PVP	Polyvinylpyrrolidone
SD	Standard deviation
SEM	Scanning electron microscopy
SPR	Surface plasmon resonance
ST	*Solanum tuberosum*
TBABr	Tetrabutylammonium bromide
TGA	Thermogravimetric analysis
Tg	Glass transition temperature
UV–Vis	Ultraviolet–visible spectroscopy
XRD	X-ray diffraction
